# A GOLD MINE OF TEXT TEXT MINING VERSUS MANUAL CODING METHODS REGARDING QUALITY OF CARE IN LONG-TERM CARE FOR OLDER ADULTS

**DOI:** 10.1093/geroni/igad104.0265

**Published:** 2023-12-21

**Authors:** Coen Hacking, Hilde Verbeek, Jan Hamers, Sil Aarts

**Affiliations:** Maastricht University, Maastricht, Limburg, Netherlands; Maastricht University, Maastricht, Limburg, Netherlands; CAPHRI, Maastricht University, Maastricht, Limburg, Netherlands; Maastricht University, Maastricht, Limburg, Netherlands

## Abstract

In long-term care for older adults, large amounts of text are collected that cover the quality of care, such as transcribed interviews, medical records, and text in electronic health records. Researchers currently analyze textual data manually to gain insights, which is a time-consuming process. Text mining (TM) could provide a solution, as this methodology can analyze large amounts of text automatically. Therefore, this study aims to compare TM to the gold standard of manual coding regarding sentiment analysis and thematic content analysis. Data was collected from interviews with residents (n=21), family (n=20) and care professionals (n=20). TM models were developed and compared to the manual approach. The results of the manual and TM approach were evaluated in three ways: accuracy, consistency and expert feedback. Accuracy showed how similar the approaches are. Consistency showed if an individual approach finds the same themes in similar text segments. Expert feedback was used to show the perceived correctness of the TM approach. The accuracy analysis showed that 81.8% of text segments receive the same sentiment code in both approaches and 83.7% of text segments receive the same thematic codes. Interviews coded by TM had a higher consistency compared to those coded manually. Expert feedback showed that the TM had a limited understanding of the context. However, it also showed certain inconsistencies of manual coding. The current study has shown that TM can be an effective tool for quickly and accurately identifying sentiment and thematic content in large amounts of textual data.

